# Combination of proton- or X-irradiation with anti-PDL1 immunotherapy in two murine oral cancers

**DOI:** 10.1038/s41598-024-62272-z

**Published:** 2024-05-21

**Authors:** Anne Marit Rykkelid, Priyanshu Manojkumar Sinha, Charlemagne Asonganyi Folefac, Michael R. Horsman, Brita Singers Sørensen, Tine Merete Søland, Olaf Joseph Franciscus Schreurs, Eirik Malinen, Nina Frederike J. Edin

**Affiliations:** 1 Department of Physics, University of Oslo, P.O. Box 1048, 0316 Blindern, Oslo, Norway; 2 Experimental Clinical Oncology - Dept. Oncology, Aarhus University Hospital, Aarhus, Denmark; 3 Danish Centre for Particle Therapy, Aarhus University Hospital, Aarhus, Denmark; 4 Institute of Oral Biology, University of Oslo, P.O. Box 1052, 0316 Blindern, Oslo, Norway; 5 Department of Radiation Biology, Oslo University Hospital, P.O. Box 4950, 0424 Nydalen, Oslo, Norway; 6 Department of Clinical Medicine, Health, Aarhus University, Aarhus, Denmark

**Keywords:** Oral cancer, Cell death and immune response

## Abstract

Combining radiation therapy with immunotherapy is a strategy to improve both treatments. The purpose of this study was to compare responses for two syngeneic head and neck cancer (HNC) tumor models in mice following X-ray or proton irradiation with or without immune checkpoint inhibition (ICI). MOC1 (immunogenic) and MOC2 (less immunogenic) tumors were inoculated in the right hind leg of each mouse (C57BL/6J, n = 398). Mice were injected with anti-PDL1 (10 mg/kg, twice weekly for 2 weeks), and tumors were treated with single-dose irradiation (5–30 Gy) with X-rays or protons. MOC2 tumors grew faster and were more radioresistant than MOC1 tumors, and all mice with MOC2 tumors developed metastases. Irradiation reduced the tumor volume in a dose-dependent manner. ICI alone reduced the tumor volume for MOC1 with 20% compared to controls, while no reduction was seen for MOC2. For MOC1, there was a clear treatment synergy when combining irradiation with ICI for radiation doses above 5 Gy and there was a tendency for X-rays being slightly more biologically effective compared to protons. For MOC2, there was a tendency of protons being more effective than X-rays, but both radiation types showed a small synergy when combined with ICI. Although the responses and magnitudes of the therapeutic effect varied, the optimal radiation dose for maximal synergy appeared to be in the order of 10–15 Gy, regardless of tumor model.

## Introduction

Radical radiotherapy of head and neck cancer (HNC) has largely remained unchanged during the last decades, with survival levels for patients with human papilloma virus (HPV) negative disease at around 50%^1^. Intensity modulated radiotherapy (IMRT) using high-energy X-rays has reduced the prevalence and severity of side effects compared to 3D conformal radiotherapy^2^, but proton therapy may further reduce toxicities^3^. In clinical practice, the relative biological effectiveness (RBE) of protons is assumed to be a constant value of 1.1 compared to X-rays^4^. However, RBE varies with proton energy and is likely dependent on tissue type and endpoint^4^. Moreover, it is an open question whether RBE changes when combining proton therapy with systemic therapies^5^.

Programmed cell death protein 1 (PD-1) is an inhibitory receptor expressed on several types of immune cells, which upon interaction with programmed death-ligand 1 (PDL1) is activated and inhibit cytokine secretion, T cell activation, and T cell proliferation and survival. Tumor cells may escape discovery and elimination by overexpression of PDL1, thus limiting the immune response^6^. Immunotherapy has emerged as a new pillar in cancer treatment, and use of immune checkpoint inhibitors (ICIs) such as anti-PDL1 has been shown to significantly prolong the survival of cancer patients across several cancer types^7–9^. It has also shown an ability to suppress tumor growth in several different murine tumor models^10–12^. However, the majority of patients with HNC do not respond to ICI monotherapy^13^, exemplified by a recent study which found a response rate of 13% to anti-PD1-treatment^14^. This may be due to lack of anti-tumor T cell activation and infiltration and/or the presence of immunosuppressive cells and cytokines^15^.

There is increasing interest in combining ionizing radiation with immunotherapy to improve outcomes, as radiation may cause immunomodulatory effects^16^. Ionizing radiation may promote the release of damage associated molecular patterns (DAMPs) that in turn attract and activate anti-tumor T cells^17,18^. However, irradiation may at the same time trigger immunosuppressive responses^19^. Protons and other ions have been suggested to be superior to X-rays in stimulating the immune system due to the differences in energy deposition patterns compared to X-rays, which has been shown for a colon cancer mouse model^20^. Still, the immunostimulatory and immunosuppressive actions of ionizing radiation may be dependent on dose, fractionation, and radiation quality^21^, and the mechanisms dependencies and impact on treatment outcomes are poorly understood.

To our knowledge, the efficacy of combined proton therapy and ICI, and comparison with X-ray therapy, has not been previously investigated. Also, to our knowledge, RBE estimates for protons in combination with ICI are generally lacking. In this study, we have assessed the effect of anti-PDL1 immunotherapy in combination with proton or X-irradiation in C57BL/6 mice with tumors from two murine oral cancer cell lines with different immunogenicity; MOC1 and MOC2. The aim was to determine if the combination of proton therapy and ICI gave a synergistic effect compared to proton therapy alone, and to compare this to X-rays with or without ICI.

## Results

MOC1 and MOC2 cells showed different histology (Supplementary Figures S1 and S2) and tumor growth characteristics when implanted in the mice. Time from inoculation to reach treatment size (150–250 mm^3^) was 6–19 days (median of 8 days) for MOC2 and 15–68 days (median of 28 days) for MOC1. Ninety-two % of MOC1 and one-hundred % of MOC2 inoculated mice successfully developed tumor in the right hind foot and were included in the experiment. For MOC1, 14/179 showed metastatic spread mainly to the lymph nodes, all more than 54 days after inoculation and 33 days after treatment start. For MOC2, all mice developed lymph node or lung metastases and had to be euthanized between day 3 and 25 (median 12 days) even if the primary tumor was reduced by the treatment. There were also a few occurrences of animals that died before they could be euthanized.

**Figure 1:**
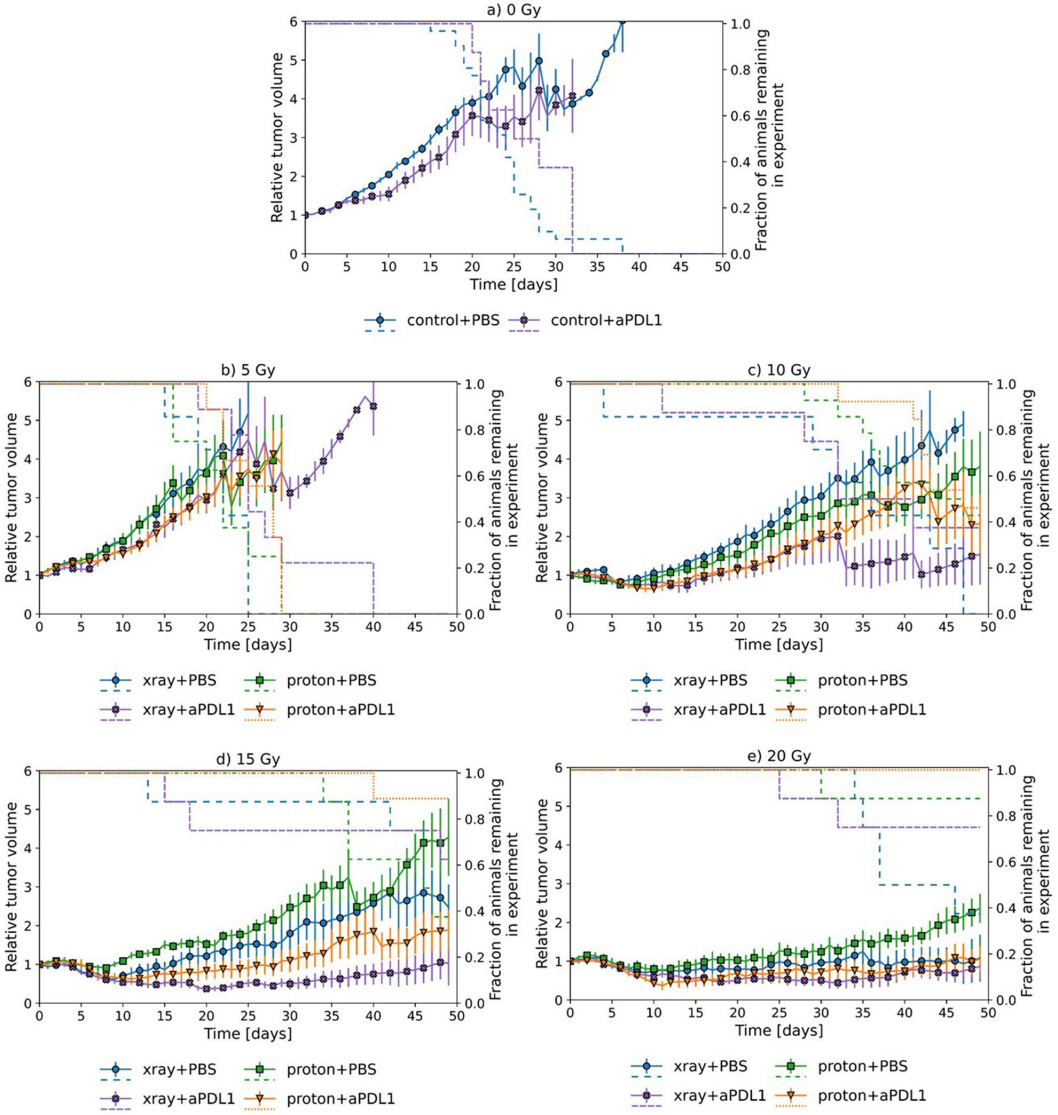
Tumor volume growth for MOC1 tumors. Mean relative volume plotted against time for MOC1 tumors (**a**–**e**) with dashed lines corresponding to surviving fraction. Treatment with and without anti-PDL1 in combination with both X-rays and protons can be seen for the different doses. (**a**) 0 Gy, (**b**) 5 Gy, (**c**) 10 Gy, (**d**) 15 Gy, (**e**) 20 Gy. Error bars correspond to 95% confidence interval.

**Figure 2:**
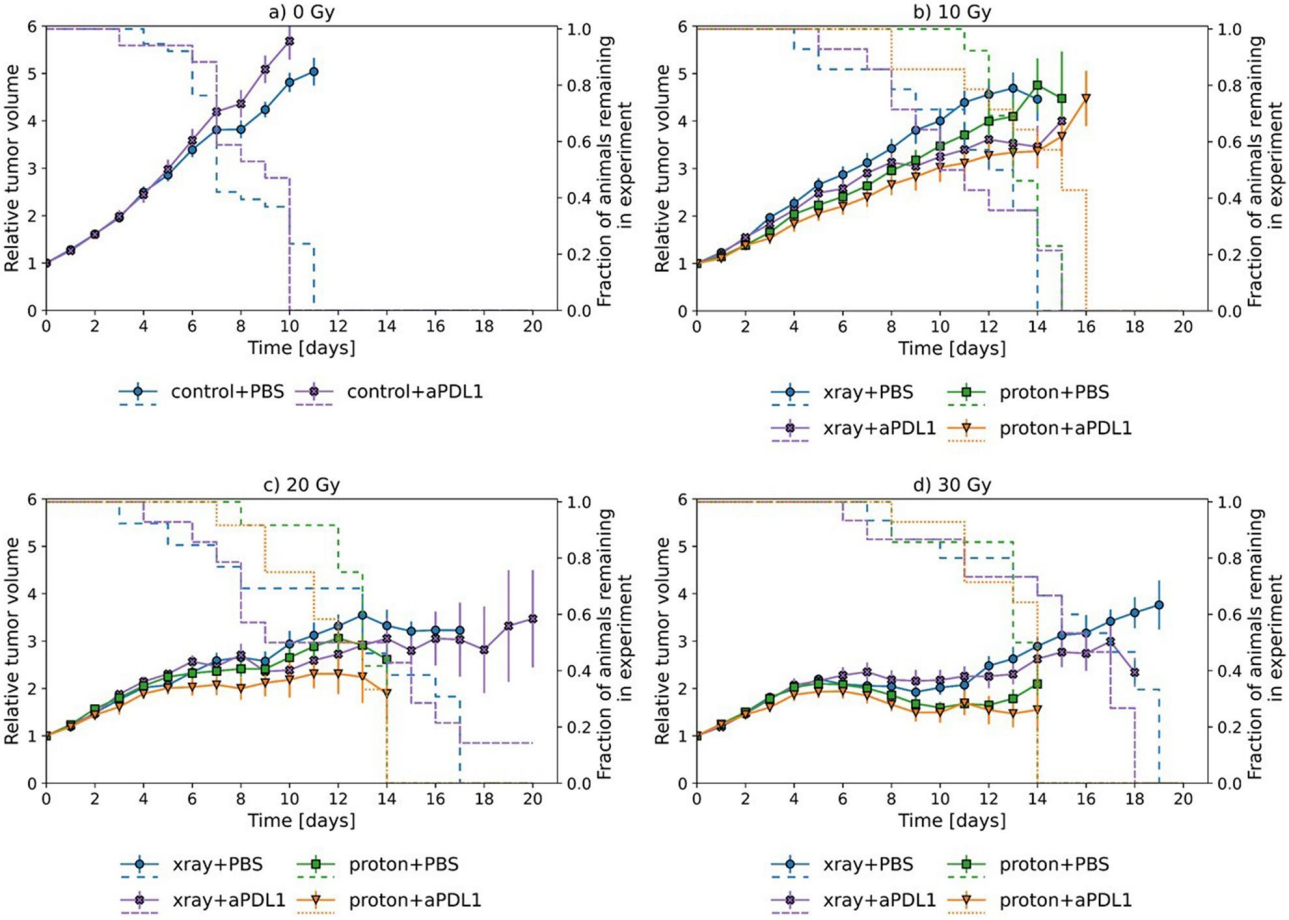
Tumor volume growth for MOC2 tumors. Mean relative volume plotted against time for MOC1 tumors (**a**–**e**) with dashed lines corresponding to surviving fraction. Treatment with and without anti-PDL1 in combination with both X-rays and protons can be seen for the different doses. (**a**) 0 Gy, (**b**) 10 Gy, (**c**) 20 Gy, (**d**) 30 Gy. Error bars correspond to 95% confidence interval.

Figures 1 and 2 show the tumor volume as a function of time after initiation of treatment for MOC1 and MOC2, respectively, together with survival curves for each treatment group. Table 1 provides estimates of the relative effect of a given treatment (mean tumor volume relative to that of a reference treatment). Comparing Figs. 1 and 2, it is seen that MOC2 grows roughly twice as fast and requires higher radiation dose for significant growth retardation compared to MOC1. Also, the rapid decline in survival for animals with MOC2 tumors indicates the aggressiveness of this model.

**Table 1:** Table shows the treatment effect as the tumor volume for a treatment relative to the tumor volume of a reference treatment, averaged over a time period (Eq. 1).

Tumor cell line	Dose	Days	aPDL1 *vs* PBS		Proton *vs* X-rays	
			Xray	Proton	PBS	aPDL1
MOC1	0	10–19	0.80 ± 0.07**			
	5	10–19	0.82 ± 0.08*	0.80 ± 0.09*	1.00 ± 0.11	0.96 ± 0.16
	10	20–29	0.61 ± 0.11**	0.72 ± 0.10*	0.85 ± 0.11	1.01 ± 0.19
	15	20–29	0.32 ± 0.06**	0.50 ± 0.09**	1.28 ± 0.21	2.03 ± 0.43**
	20	20–29	0.62 ± 0.14*	0.61 ± 0.14**	1.32 ± 0.24	1.30 ± 0.34
MOC2	0	5–9	1.11 ± 0.09			
	10	10–14	0.78 ± 0.10*	0.81 ± 0.10*	0.91 ± 0.12	0.93 ± 0.14
	20	10–14	0.84 ± 0.13	0.77 ± 0.17	0.87 ± 0.11	0.81 ± 0.20
	30	10–14	0.97 ± 0.14	0.89 ± 0.19	0.73 ± 0.11**	0.67 ± 0.13**

Uncertainties are given as 95% confidence intervals. Reference treatments are PBS or X-rays. *p*-values were calculated as Fishers combined probability test (within a time period) where individual *p*-values for each time point used in the calculation were found by paired t-test. *p*-values < .05 are marked with * and *p*-values < .01 are marked with **.

For MOC1, anti-PDL1 treatment alone significantly reduced the tumor volume to 80 ± 7% compared to sham treatment (Table 1). For MOC1, a dose of 5 Gy gave virtually no tumor response compared to sham treatment, while 10, 15 and 20 Gy resulted in pronounced growth retardation (Fig. 1). Irradiation with doses higher than 5 Gy combined with anti-PDL1 gave a marked response in MOC1 compared to irradiation or anti-PDL1 alone, where the relative effect ranged from 40 to 70% (Table 1) regardless of radiation type. The maximal relative effect appears to be around 15 Gy, although no firm conclusions can be made. There was a tendency that X-rays overall gave a stronger MOC1 response than protons, but the difference was only significant at 15 Gy with anti-PDL1.

For MOC2, anti-PDL1 treatment alone did not result in any change in the tumor volume compared to sham treatment (Fig. 2). High radiation doses were required in order to achieve a marked reduction in tumor growth. Irradiation with 10 Gy in combination with anti-PDL1 gave a significant decrease in tumor volume of around 20% compared to irradiation only for both X-rays and protons (Table 1). A weaker, non-significant effect was found at higher doses in combination with anti-PDL1. The tendency of an improved tumor response for protons compared to X-rays was observed for all dose groups with and without anti-PDL1, but the difference was only significant at 30 Gy (Table 1).

**Figure 3:**
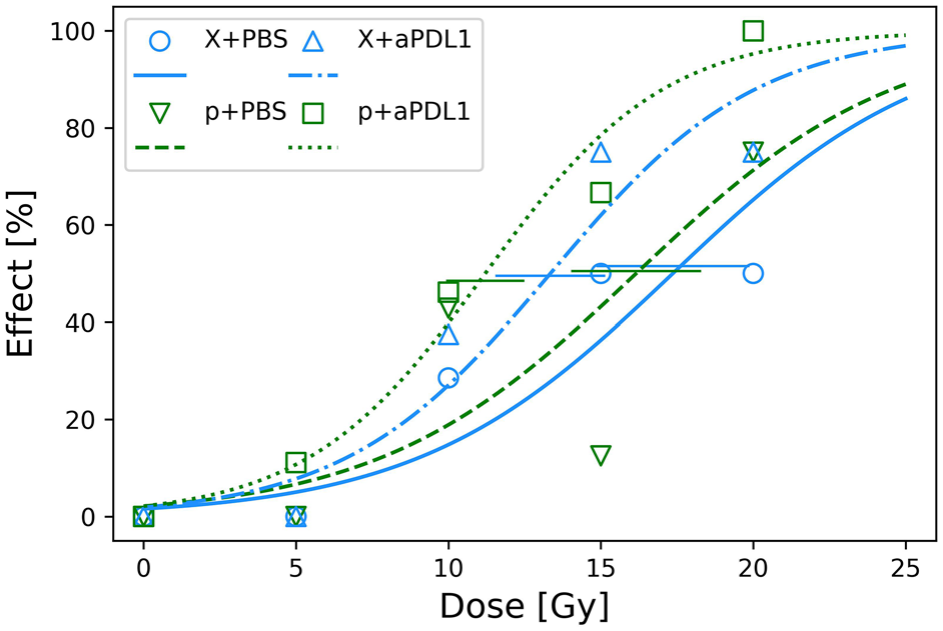
Tumor effect in terms of fraction of animals not showing progressive disease after treatment for MOC1 tumors evaluated at day 45 (Supplementary Table S3). Points show data, while lines correspond to logistic regressions on data for respective treatment groups with estimated SEM in TD_50_ as horizontal bars.

MOC1 mice with complete remission (CR) on day 45 were mainly found in treatment groups receiving anti-PDL1 with doses larger than 10 Gy. For protons, CR was achieved after 10 Gy (2/13; 15%), 15 Gy (1/9; 11%) and 20 Gy (2/8; 25%) with anti-PDL1 treatment. For X-rays, CR was achieved for 15 Gy (2/8; 25%) and 20 Gy (1/8; 13%) with anti-PDL1 and for 20 Gy alone (1/8; 13%). None of the MOC1-bearing mice with complete or partial remission developed metastases within the observation time of 3–4 months before termination. The treatment effect in terms of a given animal with permanent or temporary remission (CR, PR and TR in Supplementary Table S3) was analyzed for MOC1 tumors using logistic regression (Fig. 3). In this case, no significant differences were observed in TD_50_ values between proton and X-rays alone or when combined with anti-PDL1 treatment (Table 2). Pooling the data for protons and X-rays in combination with anti-PDL1 or PBS gave 95% CIs in TD_50_-values of (10.2, 14.2) Gy and (13.9, 20.6) Gy, respectively. The CI’s overlap, although marginally, but the remission data still indicate that the combination of irradiation and anti-PDL1 treatment is superior to irradiation alone. It is noted that this analysis was not possible for MOC2, showing extreme tumor growth and rapid metastization with virtually no true responders.

**Table 2:** TD50-values for MOC1 from logistic regression on survival levels for each treatment.

Treatment	TD_50_ (Gy)
X-rays + PBS	17.4 ± 5.0
Protons + PBS	16.2 ± 4.1
X-rays + anti-PDL1	13.3 ± 3.3
Protons + anti-PDL1	11.2 ± 2.4

## Discussion

There is great interest in combining radiotherapy and PDL1 blockade, as the efficacy of either treatment is limited for many cancers. Still, it is not clear how to optimize the combination treatment, as the outcome depends on radiation type, dose, and fractionation, among others^22^. To our knowledge, we present the first animal study assessing the efficacy of combining proton therapy with an immune checkpoint inhibitor. We show that the MOC1 and MOC2 tumors responded very differently to protons and X-rays with or without anti-PDL1-treatment. MOC1 tumors grew slower, were more radiosensitive, and showed less metastases than MOC2 tumors. ICI gave a 20% reduction in MOC1 tumor volume compared to sham treatment. Combination therapy with irradiation and ICI gave 40–70% volume reduction compared to irradiation alone for doses greater than 5 Gy, possibly peaking at 15 Gy. This indicates that there is a synergistic window for both protons and X-rays for this tumor model. Previously, fractionation doses of 10–13 Gy have been reported to obtain optimal synergistic effect with CTLA-4 treatment^23^, in line with our finding. For MOC2, a slightly higher tumor response was observed for protons compared to X-rays, although this cannot be firmly concluded. Immunotherapy alone had no effect on MOC2 tumor growth, but in combination with radiotherapy, there were indications of a synergistic effect for both X-rays and protons, conceivably peaking around 10 Gy. Seen together with the findings from MOC1, our data indicate an optimal radiation dose of 10–15 Gy to maximize the synergy with ICI.

The higher efficacy of X-rays compared to protons for MOC1 tumors, as evaluated from the tumor growth curves up to day 30, indicates that the RBE is lower than unity for this model. A proton RBE of $\sim$ 0.8 has been reported previously for tumor growth delay of NFSa fibrosarcoma cells transplanted in C3H/He mice^24^. However, using the survival data on day 45 (Fig. 3), we found that protons gave a slightly higher response in terms of a lower TD_50_, although the difference to X-rays was not significant. For MOC2 tumors, using growth data only, protons had a slightly higher effect than X-rays (only significant for 30 Gy) when given alone, indicating RBE > 1 for this model. Combining irradiation with anti-PDL1 treatment increased the response for both MOC1 and MOC2 (only significant for 10 Gy) compared to irradiation alone, but the impact was greatest for the MOC1 tumor model. Combined with anti-PDL1, the treatment efficacy for MOC1 tumors was significantly greater than for MOC2, indicating that tumor response is affected by tumor characteristics in addition to adjuvant use of immunotherapy. From H&E-stained tumor sections (Supplementary material), we found that MOC2 tumors were largely undifferentiated while MOC1 was a moderately to highly differentiated oral squamous cell carcinoma. Thus, the most differentiated tumor (MOC1) had the lowest RBE, which is in line with the findings by Glowa et al. who found that RBE for carbon ion therapy decreased with tumor differentiation for rat prostate cancers^25^. As in the present study, the increase in RBE predominantly resulted from a decrease in X-ray effect with increasing differentiation while the variation in treatment response between the rat cell lines to carbon ions was smaller.

The rationale for combining irradiation with immune checkpoint inhibitors is that inhibition of immune checkpoints only has an effect if the immune system recognizes the tumor cells as foreign. Radiation has been shown to induce immunogenic cell death, which activates anti-tumor cytotoxic T cells. The two MOC cell lines were chosen due to their different immunogenicity^26,27^ with MOC1 being immunogenic, and therefore expected to respond to immune checkpoint inhibition without irradiation. MOC2, on the other hand, is less immunogenic and in addition very aggressive^28^. Consistent with these characteristics, we found a significant reduction in MOC1 tumor size in mice treated with anti-PDL1 alone. The growth rate of untreated MOC2 tumors was much higher than for MOC1 and was not affected by anti-PDL1 treatment. In the study by Judd et al. comparing growth of MOC1 and MOC2 tumors in immunodeficient RAG2–/– mice and WT C57Bl/6 mice, MOC2 tumors grew at the same rate in immunodeficient and WT mice and generally faster than MOC1 tumors. Conversely, MOC1 tumors grew slower in WT than in immunodeficient mice^29^. In addition, an elevated CD8 + T cell infiltration in MOC1, but not MOC2 tumors, was observed, indicating that the MOC1 tumor growth was slowed down by the immune system^29^.

For MOC2, a tendency of lower tumor growth rate found for protons and X-rays in combination with anti-PDL1 compared to irradiation alone, with a reduction in tumor size of about 20% at 10 Gy. Since the MOC2 tumors are not immunogenic without irradiation and demonstrate no response to ICI alone, the synergistic effect with irradiation and anti-PDL1 suggests that this could be a way of increasing the treatment efficacy for aggressive and less immunogenic tumors. Also, proton irradiation gave consistently greater growth reductions compared to X-irradiation, which could be an ‘LET effect’ with an RBE of around 1.1. For the already immunogenic MOC1 tumors, we observed an increased, possibly synergistic, effect for both X-rays and protons combined with anti-PDL1 for doses above 10 Gy. This could indicate that the irradiation improved the already present immunogenic response or that it decreased immunosuppressive mechanisms. Since protons with anti-PDL1 tentatively gave the best outcome for MOC1 after 45 days, with the same tendency for MOC2, one could speculate whether protons are superior in inducing immunogenic responses, while X-rays may increase tumor growth delay through different mechanisms. This is partly substantiated by a study comparing radiation-induced immunogenicity for X-rays and carbon ions^30^. In that study, carbon ions gave both high levels DAMPs in the form of HMGB1 and low levels of immunosuppressive cytokines, while X-rays only gave increased HMGB1^30^.

For MOC1 tumors, a few of the treated animals developed metastases before termination of the experiment, and these mice had only temporary tumor remission. Thus, for the MOC1 cell line, metastases seemed to be a consequence of high tumor burden. All animals with MOC2 tumors developed metastases, even the mice with complete tumor response, and had to be euthanized preventing long term follow-up. This implies that metastases also developed during anti-PDL1 treatment and thus only a local, but not a systemic response was seen for MOC2 tumors. This would suggest that MOC2 tumors develop metastases with immunosuppressive characteristics, which do not respond to antitumor immune signals, even in the animals where the tumor shows a synergistic response to radiation and anti-PDL1. It also indicates that inducing an immunogenic response and blocking immune checkpoints are not sufficient to induce a response in MOC2 metastases. Thus, MOC2 appears to be an excellent model for future studies aiming at developing treatment strategies for aggressive H&N tumors that are resistant to radiation- and immune therapy.

The main limitation to this study is the number of animals in the treatment groups especially for MOC2 as well as the administration plan of ICI only lasting for 11 days with 4 injections. Due to the short lifespan of MOC2 inoculated mice, some animals did not receive all injections. Also, the injection of tumor tissue in place of laboratory grown cells may give a larger variation in number of viable tumor cells and possibly result in a less homogeneous cell population injected to inoculate the tumors.

In conclusion, we found synergistic effects from combining radiation and immunotherapy in both tumor models for both protons and X-rays, which was pronounced in the well-differentiated MOC1 but less marked in the poorly differentiated MOC2. Although the responses and magnitudes of the therapeutic effect varied, the optimal radiation dose for maximal synergy appeared to be in the order of 10–15 Gy, regardless of tumor model. In MOC2, where all animals developed metastases, it was not possible to detect a systemic treatment effect. In MOC1, high tumor effect from the treatment appeared to prevent the development of metastases. Radiation combined with ICI appears to be a potent way of boosting the effect of ICI, but our findings warrant further validation and mechanistic studies.

## Method

### Murine mouse model

Two Mouse Oral Cancer (MOC) cell lines were obtained (Kerafast, USA). MOC1 is a cell line with indolent growth in vivo and it is very immunogenic, while MOC2 is a less immunogenic, aggressive cancer with rapid growth in vivo and propensity to metastases^28,29^. C57BL/6Jrj female mice were used throughout (Janvier, France) after a minimum of 2 weeks of acclimatization in the animal facility at Danish Centre for Particle therapy, Aarhus, Denmark, where they were stalled in cages of 4 mice. The mice were around 9–18 weeks at inoculation (Supplementary Tables S1 and S2). Tumor tissue was first implanted into the mouse flank. When the tumor reached a size of approximately 1000 mm^3^ it was harvested and processed into a cell suspension in PBS, and 5–8 µl was injected subcutaneously into the right hind leg of each mouse. All animal experiments were performed in accordance with the animal welfare policy of Aarhus University and approved by the Danish Animal Experiments Inspectorate.

### Treatment and follow-up

Treatment started when the tumor size reached approximately 200 mm^3^ (range 150–257 mm^3^). The treatment groups included treatments with anti-PDL1, X-rays, protons, X-rays with anti-PDL1, and protons with anti-PDL1. Controls were injected with PBS. Four different radiation doses were used for MOC1 tumors and three for MOC2 tumors resulting in 32 treatment groups in total with at least 7 mice in each and a total of 398 mice (Supplementary Tables S1 and S2). The mice were weighted twice a week and visually inspected daily. The planned humane endpoint was 20% decline in body weight relative to baseline, but all mice with MOC2 tumors developed lymph node metastases and were euthanized due to bleeding tumors and general decline in health. Tumor volume (TV) was calculated as an ellipsoid volume from length (L), width (W) and height (H) caliper measurements as TV = π/6 × L × W × H. TV was measured every weekday after treatment until end of follow up. Mice were euthanized when the tumor reached 1000 mm^3^ or three times TV compared to day 0, or earlier if humane endpoint was reached. At euthanasia, the tumor was harvested (when possible) and immediately put in formalin buffer for fixation with duration of 24–72 H at 5 degrees Celsius before it was dehydrated and embedded in paraffin. No exclusion criteria were determined a priori, and no animals were excluded from the experiment.

An initial ICI screening with anti-CTLA4, anti-PD1, and anti-PDL1 showed no differences for MOC2 tumors. A significant growth reduction was seen for MOC1 tumors treated with anti-PDL1, but not anti-CTLA4, anti-PD1. Anti-PDL1 is a drug already approved and used in the clinic and it was selected for further experiments. In the current study, mice were injected intraperitoneally with anti-PDL1 (InVivoMAb anti-mouse PDL1 (B7-H1), Clone 10F.9G2, BioXCell, USA), stock concentration 7.95 mg/ml, 10 mg/kg mouse or with PBS on days 1, 4, 8 and 11 after irradiation.

Irradiations with both X-rays and protons were performed using the same setup. Non-anesthetized mice were restrained in a custom-designed Lucite jig, with the right foot fixed in an outstretched position on a resting plate attached to the jig. It was ensured that the foot was not clamped to avoid reduced blood supply and hypoxia. The mice were placed in a custom water tank with the foot fully emerged in water with temperature 25 °C during irradiation. The mouse body was shielded with a brass collimator and only the tumor-bearing leg was in the radiation field. For proton irradiations, the leg was placed in the middle of the spread-out Bragg peak using a horizontal scanning proton pencil-beam (ProBeam, Varian, USA). The proton irradiation procedure has been described in detail elsewhere^31–33^. X-irradiations were performed using a YXLON Maxishot X-ray unit (280 kV, 2 Gy/min) with the dosimetry controlled by a Semiflex ionization chamber (PTW, Germany). The doses given to the tumor in a single fraction at day 0 were 5, 10, 15, and 20 Gy for MOC1 and 10, 20, and 30 Gy for MOC2. Mice were treated when the tumor size reached 200 (150–257) mm^3^ and were randomized to different treatments and doses, researchers were not blinded, and potential confounders were not controlled.

Mice with MOC2 tumors were euthanized due to metastases typically 6–19 days after treatment, and it was not possible to access long-term effects. Response categories were defined in order to estimate long-term treatment effect for MOC1 tumors. Cutoff was chosen at day 45 after treatment for MOC1 tumors. The categories were: (1, CR) Complete Remission = No trace of primary tumor, (2, PR) Partial Remission = Primary tumor smaller than at start of treatment, (3, TR) Temporary Response = Primary tumor shrinks to smaller size than at start of treatment and stays below that size for 5 days or more but with significant regrowth, and (4, PD) Progressive disease = Mouse lives shorter than the cutoff day and/or has a continually growing primary tumor.

### Data analyses

All analyses were done in Python v3.8.8 with libraries NumPy, SciPy, seaborn and statsmodels. Error bars represent 1 standard error of the mean (SEM) or 95% confidence interval (stated for individual purposes).

As tumor volumes were not measured every single day, volumes for missing days were estimated by linear interpolation from the closest measurements. Tumor volumes were normalized to measurements on day 0 (day of treatment start). Mean values of relative tumor volume on each day were calculated from all mice with the same tumor receiving the same treatment as there were only small variations between separate experiments.

To test for significant differences between treatment groups, a two-sided t-test for tumor volumes on individual days was used. Based on those results, Fisher's combined probability test for a time period was used to find a *p*-value reflecting differences between treatments within the given period. As an estimate of the reduction in tumor size due to a treatment during this period a comparative analysis was performed. Here, we calculated the mean tumor volume for a given treatment relative to a reference for the same period (Eq. 1).

1 $${E}_{treatment}(t1-t2)=\left(\sum_{i=t1}^{i=t2}\frac{T{V}_{treatment}(i)}{T{V}_{reference}(i)}\right)/\left(t2-t1\right),$$

where t1 is the first day and t2 is the last day included in the analysis, TV is the mean relative tumor volume and E is the effect. To avoid selection bias, time periods were chosen to have similar high surviving fractions in compared groups.

Tumor response data in terms of binary treatment effect were analyzed using logistic regression and subsequent estimate of the tumor dose giving 50% effect (TD_50_). Effect was defined as permanent or temporary remission (CR, PR or TR) while no effect was defined as progressive disease (PD).

### Supplementary Information

Supplementary Information.

## Data Availability

Research data are stored in an institutional repository and will be shared upon request to the corresponding author.
