# Author Correction: Combination of proton- or X-irradiation with anti-PDL1 immunotherapy in two murine oral cancers

**DOI:** 10.1038/s41598-024-66148-0

**Published:** 2024-07-03

**Authors:** Anne Marit Rykkelid, Priyanshu Manojkumar Sinha, Charlemagne Asonganyi Folefac, Michael R. Horsman, Brita Singers Sørensen, Tine Merete Søland, Olaf Joseph Franciscus Schreurs, Eirik Malinen, Nina Frederike J. Edin

**Affiliations:** 1 Department of Physics, University of Oslo, Blindern, P.O. Box 1048, 0316 Oslo, Norway; 2 Experimental Clinical Oncology - Dept. Oncology, Aarhus University Hospital, Aarhus, Denmark; 3 Danish Centre for Particle Therapy, Aarhus University Hospital, Aarhus, Denmark; 4 Institute of Oral Biology, University of Oslo, Blindern, P.O. Box 1052, 0316 Oslo, Norway; 5 Department of Radiation Biology, Oslo University Hospital, Nydalen, P.O. Box 4950, 0424 Oslo, Norway; 6 Department of Clinical Medicine, Health, Aarhus University, Aarhus, Denmark

Correction to: *Scientific Reports* 10.1038/s41598-024-62272-z, published online 21 May 2024

The original version of this Article contained errors in the Supplementary Information file, where the Author information was incorrect, and the legend of Supplementary Figure S2 was incomplete.

The original incorrect Supplementary Information file is provided below.

The Supplementary Information file that accompanies the original Article has been corrected.

### Supplementary Information

Supplementary Information.

