# Supplementary material for: Combination of proton- or X-irradiation with anti-PDL1 immunotherapy in two murine oral cancers
Source: Sci Rep. 2024 May 21;14:11569. doi: 10.1038/s41598-024-62272-z (PMC11109162; doi:10.1038/s41598-024-62272-z)
Supplement: Supplementary file 1 — Supplementary Information. [file 41598_2024_62272_MOESM1_ESM.pdf]

Combination of proton- or X-irradiation with anti-PDL1 immunotherapy in two murine oral cancers

Short Running Title: *Synergies of protons and ICI*

Author Names

Anne Marit Rykkelid[1], Priyanshu Manojkumar Sinha[2], Charlemagne Asonganyi Folefac[2], Michael R. Horsman[2], Brita Singers Sørensen[2,3,4], Tine Merete Søland[5], Olaf Joseph Franciscus Schreurs[5], Eirik Malinen[1,6†], Nina Frederike J. Edin[1†]*

Author Institutions

[1]*Department of Physics, University of Oslo, PO Box 1048 Blindern, 0316, Oslo, Norway*

[2] *Experimental Clinical Oncology - Dept. Oncology, Aarhus University Hospital, Aarhus, Denmark*

[3]*Danish Centre for Particle Therapy, Aarhus University Hospital, Palle Juul-Jensens Blvd. 99, 8200 Aarhus, Danmark*

[4]*Department of Clinical Medicine, Health, Aarhus University.*

[5]*Institute of Oral Biology, University of Oslo, PO Box 1052 Blindern, 0316 Oslo, Norway*

[6]*Department of Radiation Biology, Oslo University Hospital, P.O. Box 4950 Nydalen, 0424 Oslo, Norway.*

[†] *Joint senior authors.*

***Corresponding Author** *Nina Frederike J Edin, n.f.j.edin@fys.uio.no, Department of Physics, University of Oslo, PB. 1048, 0316 Blindern, Norway*

Supplementary material

Supplementary table S1. Number of experimental animals in individual MOC1 experiments and treatment groups. N is the number of experiments for each treatment group and n is the total number of animals in each group. The number of animals and age at inoculation for each experiment is given in the bottom rows.

MOC1			**Experiments**						
	**Dose (Gy)**	**Drug**	**1**	**2**	**3**	**4**	**5**	**N**	**n**
**Controls**	**0**	**PBS**	7	7	13	4		4	31
		**PD-L1**	8					1	8
**Proton**	**5**	**PBS**		4	4			2	9
		**PD-L1**		5	4			2	9
	**10**	**PBS**	7	4	3			3	14
		**PD-L1**	7	4	2			3	13
	**15**	**PBS**		5	3			2	8
		**PD-L1**		5	4			2	9
	**20**	**PBS**		5	3			2	8
		**PD-L1**		5	3			2	8
**Xray**	**5**	**PBS**				3	4	2	7
		**PD-L1**				4	5	2	9
	**10**	**PBS**				3	4	2	7
		**PD-L1**				3	5	2	8
	**15**	**PBS**				3	5	2	8
		**PD-L1**				4	4	2	8
	**20**	**PBS**				4	4	2	8
		**PD-L1**				4	4	2	8
#Animals in experiment			29	44	39	32	35		
Age at inoculation (weeks)			23	11	9	16	18		

Supplementary table S2. Number of experimental animals in individual MOC2 experiments and treatment groups. N is the number of experiments for each treatment group and n is the total number of animals in each group. The number of animals and age at inoculation for each experiment is given in the bottom rows.

MOC2			**Experiments**							
	**Dose (Gy)**	**Drug**	**1**	**2**	**3**	**4**	**5**	**6**	**N**	**n**
**Controls**	**0**	**PBS**	9	6	9		5	9	5	38
		**PD-L1**	7	6		4			3	17
**Proton**	**10**	**PBS**		8	5				2	13
		**PD-L1**		9	5				2	14
	**20**	**PBS**			5	7			2	12
		**PD-L1**			5	7			2	12
	**30**	**PBS**			5	9			2	14
		**PD-L1**			7	7			2	14
**Xray**	**10**	**PBS**					6	8	2	14
		**PD-L1**					6	8	2	14
	**20**	**PBS**					6	7	2	13
		**PD-L1**					6	8	2	14
	**30**	**PBS**					6	9	2	15
		**PD-L1**					6	9	2	15
#Animals in experiment			16	29	41	34	41	58		
Age at inoculation (weeks)			12	28	10	13	14	11		

Supplementary table S3. Treatment effect on day 45 for MOC1 tumors. All mice were categorized with either Progressive Disease (PD), Temporary Remission (TR), Partial Remission (PR) or Complete Remission (CR) as defined in the methods section.

**Treatment**		**Treatment Effect (TE)**				
**Modality**	**IP Injections**	**Dose (Gy)**	**PD**	**TR**	**PR**	**CR**
	PBS	0	31/31			
	aPDL1	0	8/8			
**X-rays**	PBS	5	7/7			
		10	5/7	2/7		
		15	4/8	3/8	1/8	
		20	4/8	3/8		1/8
	aPDL1	5	9/9			
		10	5/8	2/8	1/8	
		15	2/8	3/8	1/8	2/8
		20	2/8	1/8	4/8	1/8
**Protons**	PBS	5	8/8			
		10	8/14	5/14	1/14	
		15	7/8	1/8		
		20	2/8	5/8	1/8	
	aPDL1	5	8/9	1/9		
		10	7/13	4/13		
		15	3/9	4/9	1/9	1/9
		20		5/8	1/8	2/8

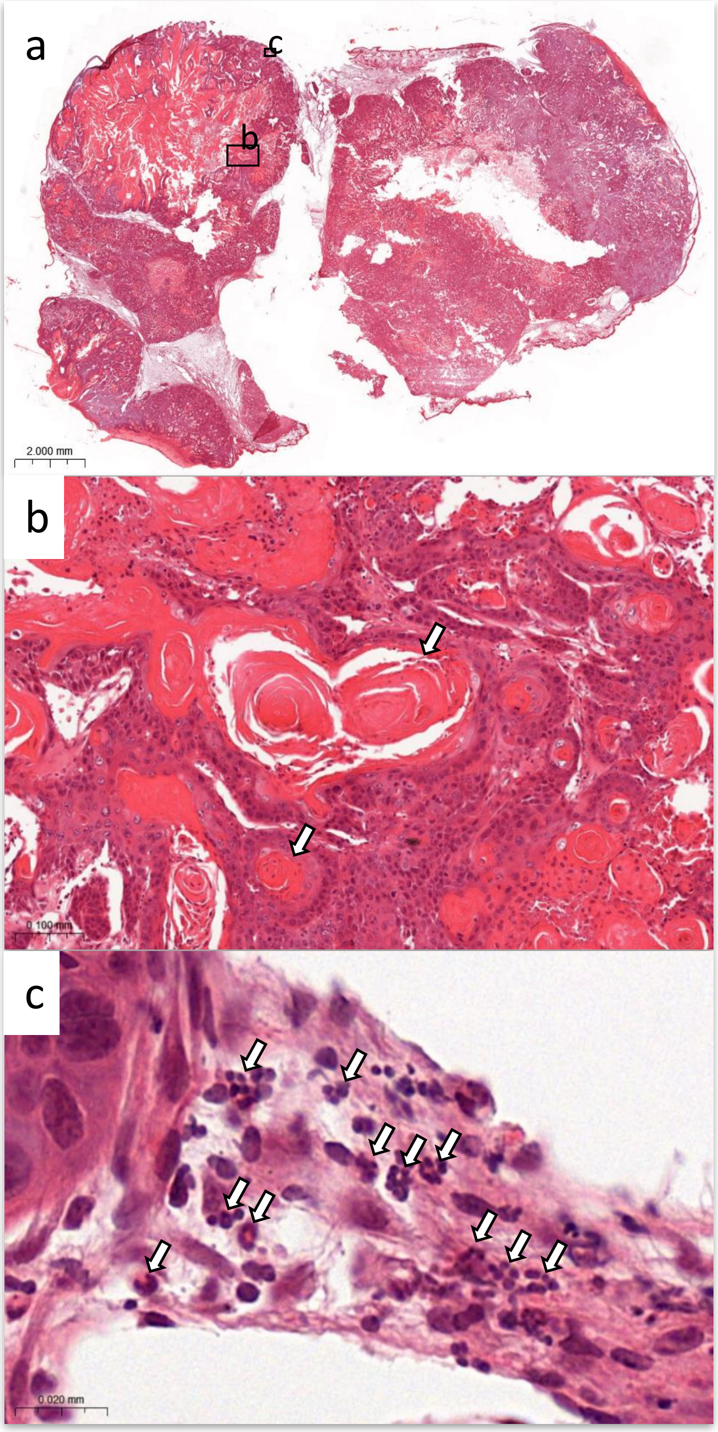

Supplementary figure S1. Histological section of hematoxylin and eosin stained (as described in the method below) untreated MOC1 tumor tissue on day 41. a) A section of the whole tumor, where the tumor is cell rich with focal areas of keratinization (b, arrows). Small bands/areas of connective tissue are seen, both within and in the periphery of the tumor tissue where a few chronic inflammatory cells are identified. Neutrophil granulocytes are present, particularly in the peripheral part of the tumor (c, arrows).

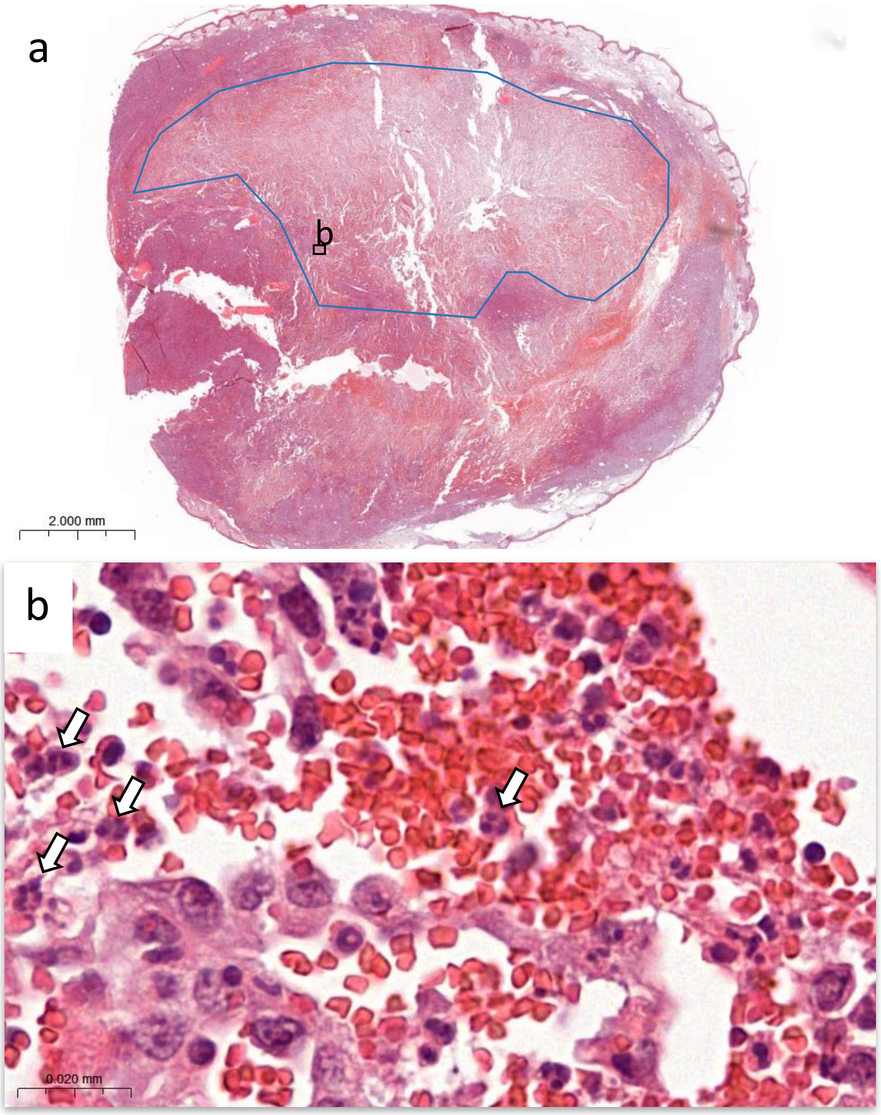

Supplementary figure S2. Histological section of a hematoxylin and eosin stained (as described in the method below) untreated MOC2 tumor on day 12. a) A section of the whole tumor is shown. The tumor tissue is disintegrated with large areas of necrotic (area marked with blue line) and partly necrotic tissue. b) The partly necrotic tissue is infiltrated by neutrophil granulocytes (arrows) and extravascular erythrocytes are seen throughout the tumor tissue.

Materials and methods - Hematoxylin and eosin staining

Four micron thick paraffin sections were mounted on object slides, dried for 1 h at 60 °C, deparaffinized in two changes of xylene for 5 min each and rehydrated in two changes of absolute ethanol and 96 % ethanol, and finally 70 % ethanol for 2 min each before washing in tap water for 5 min. Nuclei were stained for 1 min with hematoxylin (Shandon, Thermo Fisher Scientific, Waltham, MA, USA), washed in tap water for 5 min and blued in 0.25 % w/v hexamethylenetetramine (Prolabo, Fontenay-sous-Bois, France) for 3 min. After washing in tap water for 5 min, cytoplasm was stained using 0.5 % w/v Eosin-Yellow (Chroma, Münster, Germany) for 4 min before the slides were rinsed in tap water, quickly dehydrated through graded alcohols, cleared in xylene and cover glass mounted with Histokitt (Karl Hecht, Sondheim, Germany).
